# A study on sexual violence among women in Northern Ethiopia's 2022 conflict: mixed methods

**DOI:** 10.3389/fgwh.2024.1340038

**Published:** 2025-01-07

**Authors:** Atitegeb Abera Kidie, Seteamlak Adane Masresha, Birtukan Gizachew Ayal, Kindie Mekuria, Tsion Kokeb Kodo, Abayneh Tunta Boye, Misganaw Guadie Tiruneh, Fassikaw Kebede Bizuneh, Eneyew Talie Fenta

**Affiliations:** ^1^ Department of Public Health, College of Health Science, Woldia University, Woldia, Ethiopia; ^2^ Department of Nursing, College of Health Science, Woldia University, Woldia, Ethiopia; ^3^ School of Medicine, College of Health Science, Woldia University, Woldia, Ethiopia; ^4^ Department of Health Systems and Policy, Institute of Public Health, College of Medicine and Health Sciences, University of Gondar, Gondar, Ethiopia; ^5^ Department of Public Health, College of Medicine and Health Science, Injibara University, Injibara, Ethiopia

**Keywords:** women, sexual violence, North Wollo Zone, Ethiopia, conflict

## Abstract

**Background:**

Violence against women is both a human rights violation and a significant reproductive health issue, causing substantial morbidity. It's a pervasive global public health concern, particularly prevalent in developing regions like sub-Saharan Africa. Ethiopia faces this issue extensively despite its preventable nature, persisting as a significant challenge within the country.

**Objective:**

The study aimed to identify the extent and factors associated with sexual violence among women, children, and adolescent girls during the 2022 armed conflict in Northern Ethiopia.

**Method:**

A community-based cross-sectional study combined quantitative and phenomenological methods. We used multistage and snowball sampling, involving 574 individuals along with 10 in-depth interviews and 3 focus group discussions (FGDs). Statistical analysis relied on Stata version 16 and open code version 4.03. Quantitative analysis employed multivariable binary logistic regression, while qualitative data underwent thematic analysis.

**Result:**

The study found a 9.76% prevalence of sexual violence, with 2.4% experiencing rape during the conflict. Prostitutes faced a fourfold increased risk (AOR: 4.2, 95% CI: 1.3, 10.9). Living in areas with attacks raised the risk 2.7 times (AOR: 2.7, 95% CI: 1.1, 6.2), and a monthly income of 2,001–4,000 ETB increased it 2.5 times (AOR: 2.5, 95% CI: 1.1, 5.7). The impacts included psychosocial effects, stigma, and fear of humiliation, divorce, and displacement.

**Conclusion:**

Approximately one in ten women experienced sexual violence during the conflict. Factors such as being a prostitute, having a lower income, and living in attacked villages were significant predictors of this violence. The main impacts included psychosocial effects, external blame, and stigma, fear of humiliation, divorce, and displacement.

## Introduction

Violence encompasses any unwanted harm rooted in gender differences (1). It spans sexual abuse, physical assault, and psychological harm targeted at women (2, 3). Violence against women (VAW) is a profound violation of their human rights (4–8). The World Health Organization (WHO) defines sexual violence as any unwanted sexual act; attempt to obtain a sexual act, unwanted sexual comments or advances, or acts of sexual trafficking or other actions directed against a person's sexuality, using coercion regardless of the relationship between the perpetrator and the victim, and can occur in any setting, including the home and workplace (9). Other sources define sexual violence as any physical, psychological violence carried out through sexual means or rape, attempted rape, molestation, sexual slavery, being forced to undress or being stripped of clothing, forced marriage and insertion of foreign objects into the genital opening or anus, forcing individuals to perform sexual acts on one another or harm one another in a sexual manner, or mutilation of a person's genitals (10).

Recently recognized in medicine, it stands as a leading, preventable cause of morbidity and mortality (11). Globally, sexual and gender-based violence, especially in conflict zones, is a prevalent and alarming issue (12, 13). Conflict-related sexual violence refers to rape, sexual slavery, forced prostitution, forced pregnancy, forced abortion, enforced sterilization, forced marriage and any other form of sexual violence of comparable gravity perpetrated against women, men, girls or boys that is directly or indirectly linked to a conflict (14). Conflict related sexual violence consisted to violations perpetrated by armed actors such as state militaries, rebel groups, and government militias during periods of conflict or immediately post conflict. However, it excludes violations by civilian actors, such as intimate partner sexual violence or sexual crimes (15). There is a relationship between former existing patterns of gender inequality and the occurrence of widespread and systematic sexual violence in armed conflicts (16). Sexual violence is a profoundly harmful and traumatic experience that can have far-reaching psychological and social impacts on the victim, regardless of their gender. The effects of sexual violence extend beyond the individual and can have significant implications across different aspects of the victim's life (17). The prevalence of sexual violence in times of conflict can be seen as a reflection of the social attitudes towards women in peacetime; the difference is only in quantity, intensity and visibility (18). Most recently, global estimates showed that 35% of women experience some form of sexual or physical intimate or non-partner violence over the course of their lives (7). Non-partner sexual violence by male relatives, friends, acquaintances or strangers is globally common form of violence against women (19). Sexual violence is a severe threat faced by women worldwide, both from intimate partners and others (20). The prevalence of sexual violence seems to be higher in poor countries. The presence of widespread political violence and unrest appears to create an environment that enables and exacerbates the occurrence of sexual violence in Sub-Saharan African countries (21). In countries like Ethiopia, violence against women is a significant public health concern, with prevalence ranging from 19.6% to 78% (22).

Sexual violence as a weapon of war is a problematic analysis, as it overlooks significant underlying sociocultural, political, legal and socioeconomic factors that contribute to the issue (23). It is about a means of pursuing military, political goals and used as tools to defeat the enemy (24). It is a tactic of war by armed groups to terrorise, humiliate and destroy communities (18). War provides the opportunity for widespread rape as an effective strategy for ethnic cleansing (25). Conflict can result in higher levels of gender-based violence against women and girls, including arbitrary killings, torture, sexual violence and forced marriage (26). The form of sexual violence varies across conflicts, armed groups within conflict, and units within armed groups, including rape of women girls, men and boys, sexual torture, forced pregnancies, and abortion (25). Conflict related sexual violence has been a consistent feature of the conflict and perpetrators from both sides have committed attacks of sexual violence whilst occupying territory (27). In 2021, one of the bloodiest civil wars in history in Northern Ethiopia drew international attention. There was looting of medical institutions and private property, mass rapes, gang rapes, physical attacks, and verbal assaults against women (28). The Amhara region has experienced an increase in civil unrest in the last several years, including violent attacks on civilians (29). During conflict, women and marginalized groups are often the most negatively affected (14). In conflict settings, rape and sexual violence are used as strategic, systematic, and calculated tools of war, ethnic cleansing, and genocide. Wartime rape and sexual violence take different forms. These include rape, sexual slavery, forced prostitution, forced pregnancy, forced sterilization/abortion, sexual mutilation, and sexual torture. During these times, the Tigray forces made tremendous human and infrastructure destructions in these part of the country, such as summary killings, kidnapping, rape, gang rape, sexual assault of women and girls, and other severe sexual acts of violence that are considered war crimes and crimes made against humanity (30).

During conflicts, sexual violence might even be used for ethnic cleansing rape and constitute a formidable weapon of war (31, 32). Preventing violence against women involves a range of strategies, yet it persists as a significant obstacle (33), hindering the achievement of Sustainable Development Goal three. The SDGs aim to eradicate all forms of violence against women by 2030, recognizing the deep-seated impact of this issue (34). There is an increasing attention to research on conflict-related sexual violence and despite efforts, sexual violence against women endures globally, particularly in conflict zones where it's wielded as a weapon. Conducting studies, especially during conflicts, is crucial to understanding this issue and prioritizing it for intervention. Investigating violence against women, especially during conflicts, is paramount as it's often used as a tool of warfare.

The study focused on a conflict affected zone because there was conflict lasting for several months and rape might have been used as a tool of genocide. The study main aim was to uncover the scale and factors behind conflict related sexual violence during the 2022 Northern Ethiopia conflict. This research is crucial for identifying any violence against women, aiding interventions, and offering evidence to health organizations and professionals. The findings align with SDG targets, serving as a guide for programmers, policymakers, and researchers in developing violence prevention interventions.

## Methodology

### Study area, design and period

The study was conducted in North Wollo Zone, part of the Amhara region in Northern Ethiopia, with a population of about 1.5 million. It covered three areas: Mersa, Kobo, and Lalibela, from May 14 to June 2, 2022, focusing on conflicts occurring from October, 2021 to May 2022. Using a mixed design, it employed a phenomenological approach in the qualitative part to deeply understand victims' experiences. The quantitative approach is essential to generalize this study finding, while the qualitative approach was used to provide context. The mixed-methods approach synergistically combines these strengths and enhances the validity and applicability of the study finding. This study considers the necessity of employing mixed approach to address the aim of the study.

### Study population, sample size and sampling technique

The study encompassed women, children, and adolescent girls in Kobo, Lalibela, and Mersa towns within North Wollo Zone of Amhara region. A woman who was severely ill and not present during the conflict was excluded from the study. For quantitative study, the sample size was determined using single population proportion formula using level of significance at 95% confidence level (1.96), the proportion of women who experienced violence in Dessie town (22.4%) (35), 5% margin of error, a design effect of 2, and a 10% non-response rate, then the final sample size estimated for this study was 590. The sampling technique employed for quantitative study was a multi-stage sampling Proportional allocation and systematic sampling were applied to select households from chosen kebeles in Mersa, Kobo, and Lalibela. The study population comprised women, children, and adolescent girls in these areas, randomly selecting one woman from households with multiple eligible participants.

For the qualitative part, snowball sampling technique was used and the sample size for the qualitative study was guided by the degree of information saturation 10 in-depth interviews and three focused group discussions were conducted based on information saturation. FGD members included were women working with women and children at kebeles, representatives from 1 to 5 women associations, kebele administrators, and health extension workers.

### Study variables

#### Dependent variable

Sexual violence was determined based on a yes or no category. It included instances where a person was coerced into sexual intercourse without physical force, forced physically into intercourse against their will, or made to perform a degrading or humiliating sexual act. The assessment of sexual violence was based on responses to these specific questions.

#### Independent variables

Demographic factors: Age, religion, education, residence, marital status, family's monthly income, family size, occupation, sex of household head.

Individual and community factors: Media exposure, involvement in prostitution, village attacks, traumatic events during conflict.

#### Operational definition

Violence refers to any gender-based act causing physical, sexual, or psychological harm to women, including threats, coercion, or arbitrary deprivation of liberty, whether in public or private settings (22).

Non-intimate partner sexual violence (NIPV) involves sexual acts, coercion, or attempts against a person's sexuality by someone who is not an intimate partner. This can include acts committed by armed actors, family, community members, teachers, or other known or unknown individuals (36).

Physical violence encompasses actions such as slapping, punching, kicking, pushing, stabbing, shooting, and burning (5, 6, 37).

Sexual Violence: insisted on having sexual intercourse even when she did not want to but did not use physical force, physically forced her to have sexual intercourse even when she did not want to, and insisted her to do any sexual act that she felt to be degrading and humiliating (37).

Psychological/emotional violence: being insulted, or made her feel bad about herself, humiliated in front of others, having things done to scare or intimidate her on purpose, or receiving verbal threats to hurt her or someone she cared about (6, 37).

Media exposure: watching television (TV), listening to radio and reading newspaper at least once a week (38).

Trauma: In this study trauma includes injuries such as fractures, lacerations by gun, stone, sticks and burns.

#### Data collection process and quality control

The data was collected by using interviewer administered structured questionnaire for quantitative study. The survey data was collected by a team of six health extension workers, and women working on gender-related issues. These data collectors were specifically chosen based on their prior experience working with survivors of sexual violence. These health extension workers and gender workers had been selected as the focal points for reporting on gender-based violence in the aftermath of the conflict. Given the highly sensitive nature of the topic of sexual violence, the researchers took steps to properly train the data collectors. This included providing training on effective interviewing techniques for sensitive subjects, educating them on the importance of building trust and rapport with participants, and instructing them on maintaining participant confidentiality and privacy throughout the data collection process.

The careful selection of experienced, community-embedded data collectors, combined with the training they received, was intended to facilitate an ethical and trauma-informed approach to gathering this sensitive information from study participants. To keep privacy of participants, data collection was done at selected and secured places and confidentiality of information was kept anonymous in every step of the study.

During data collection, those individual insisted on having sexual intercourse even when she did not want to but did not use physical force, physically forced her to have sexual intercourse even when she did not want to, and insisted her to do any sexual act that she felt to be degrading and humiliating was considered as sexual violence victim. To ensure the data quality pretest was done on 5% of the sample.

For qualitative part, data was collected by focus group discussion guide and semi-structured questionnaires. The investigators of this study conducted both the interview and FGD. The interview and FGD was undertaken within a range of 30–45 min. Interviews and FGD was audio-recorded.

#### Data processing and analysis

Data completeness was ensured before entry into EPI data version 4.6. Statistical analysis using Stata version 16 involved managing missing data through imputation for applicable variables. Descriptive statistics were presented using text, and tables. In bivariaable analysis, variables with *p*-values ≤ 0.25 were included in multivariable analysis. Multivariable logistic regression was used to assess associations. The model goodness of fit was assessed by Hosmer and Lemeshow test and the model was well-fitted with *p*-value of 0.61. The strength of association was measured with AOR and 95% CI. Variables in this regression were considered statistically significant at a *P*-value < 0.05.

In the qualitative study, audio recordings and notes from interviews and FGDs were done and the recorded data were transcribed verbatim into Amharic language. The transcribed data also later translated into English language by the investigators. The analysis was conducted with Open Code version 4.03. Data were entered into the software. Initial coding is conducted, where segments of data are labeled with codes that represent key themes or concepts. Codes are refined and grouped into broader themes. Therefore, a thematic analysis approach was utilized to examine the data, resulting in the development of themes. The analysis involves peer debriefing to ensure accuracy and credibility. If the disagreement that arises between the authors was resolved by engage in discussions to present differing views and the consensus-building techniques was used to arrive at an agreement.

## Results

### Quantitative result

#### Socio demographic characteristics of study participants

A total of 574 women, children, and adolescent girls participated, achieving an overall response rate of 97%, mostly from rural areas. In terms of monthly income, participants earning <2,000, 2,000–3,999, and ≥4,000 were 24.22%, 51.57%, and 24.22% respectively. The mean age was 32.41 (SD ± 13.82) years. Most sexually violated women were aged 18 and above (93.2%), and 35.54%, 39.72%, 16.38%, and 8.36% were single, married, divorced, and widowed respectively. Nearly half had no formal education, accounting for 42.68% of respondents (Table 1).

**Table 1:** Socio-demographic characteristics of study participants.

Variables	Category	Frequency	Percent
Religion	Orthodox	450	78.4
	Muslim	112	19.51
	Other	12	2.03
Age	<18	39	6.8
	≥18	535	93.2
Marital status	Single	204	35.54
	Married	228	39.72
	Divorced	94	16.38
	Widowed	48	8.36
Educational status	No formal education	245	42.68
	Primary	111	19.34
	Secondary and above	218	37.98
Sex of head of household	Male	327	56.97
	Female	247	43.03
Family size	<5	293	51.05
	≥5	281	48.95

#### Individual and community characteristics of respondents

Of all participants, 5.57% were prostitutes, with over a quarter experiencing sexual violence (28.1%). The majority (73%) encountered village attacks, while 85.19% suffered physical trauma, including fractures and injuries caused by guns, stabbings, and other means (Table 2).

**Table 2:** Individual and community characteristics of study participants.

Variables	Category	Frequency	Percent
Exposure to media	Yes	128	22.30
	No	446	77.70
Prostitution	Yes	32	5.57
	No	542	94.43
Attacks in the village during war	Yes	419	73
	No	155	27
Trauma	Yes	489	85.19
	No	85	14.81

#### Prevalence of sexual violence

The prevalence of sexual violence was 9.78%, with 16.8% in Kobo, 8.6% in Mersa, and 2.4% in Lalibela. Specifically, 2.4% of women were raped.

#### Determinants of sexual violence

The study found that women involved in prostitution were four times more likely to experience sexual violence (AOR: 4.214, 95% CI: 1.268, 10.907). Additionally, sexual violence was 2.67 and 2.5 times higher among those residing in areas with village attacks (AOR: 2.67, 95% CI: 1.139, 6.237) and with a monthly income of 2,001–4,000 ETB (AOR: 2.548, 95% CI: 1.131, 5.739) respectively (Table 3).

**Table 3:** Determinants of sexual violence among women in North Wollo zone, north east Ethiopia, 2022.

Variables	Variable category	Sexual violence		COR (95% CI)	AOR (95% CI)
		No	Yes		
Prostitution	Yes	23 (71.1)	9 (28.1)	4.12 (1.80, 9.42)*	4.21 (1.63, 10.91)*
	No	495 (91.3)	47 (8.7)	1	1
Educational status	No education	217 (88.6)	28 (11.4)	1	1
	Primary	101 (91)	10 (9.0)	0.77 (0.36, 1.64)	0.92 (0.41, 2.07)
	Secondary and above	200 (91.7)	18 (8,3)	0.69 (0.37, 1.20)	0.87 (0.43, 1.76)
Attack on village	Yes	370 (88.3)	49 (11.7)	2.80 (1.24, 6.32)*	2.66 (1.14, 6.24)*
	No	148 (95.5)	7 (4.5)	1	1
Family size	<5	268 (91.5)	25 (8.5)	1	1
	≥5	250 (89.0)	31 (11.0)	1.33 (0.76, 2.31)	1.27 (0.71,2.30)
Income in ETB	<2,000			0.61 (0.19, 1.92)	0.55 (0.17, 1.81)
	2,001–4,000			2.78 (1.27, 6.09)*	2.55 (1.13, 5.74)*
	>4,000			1	1
Media exposure	Yes	117 (91.4)	11 (8.6)	1	1
	No	401 (89.9)	45 (10.1)	1.19(0.59, 2.38)	1.32(0.64, 2.73)

COR, crude odds ratio; AOR, adjusted odds ratio.

* *p* < 0.05, 1- reference.

##### Qualitative findings

The quantitative findings of the study were supported by the qualitative findings. The qualitative component of the study included in-depth interviews with ten participants who had experienced rape, from the areas of Kobo, Mersa, and Lalibela. Additionally, three focused group discussions were conducted. Through the qualitative analysis, various themes were identified based on the participants' expressions and perspectives. These qualitative themes and participant views were used to further substantiate and provide deeper context to the quantitative findings of the study. During the northern conflict, Federal government forces, TPLF, Amhara region Special Forces and Minisha were participated but the perpetrators of sexual assault in North Wollo zone was TPLF forces targeted against Amhara people as a weapon of war.

### Place of sexual assault, risky areas and conditions for exposure

The assaults occurred in diverse locations: home, roads, private workplaces, public areas, and closed houses. Among the in-depth interview participants, seven out of ten reported being raped at their homes.

A 17 year's old women said that “I was raped while I was in my house”. Similarly other 25 years old women said that “I was raped while I was alone at home”. While others stated that “two girls were raped in public”. Participant 5 from Kobo FGD supported the previous idea that “At that time three children were raped on the road, not at home”.

Prostitution areas and Amhara Special Forces' residence zones were identified as high-risk locations for sexual violence by rebels or TPLF forces. The health extension worker reported that, “Armed groups entered the house and raped women, claiming it was Minisha's residence.”

Another participant in the FGD mentioned*, “The events might not have happened uniformly everywhere. Sexual assaults by rebels mainly occur where prostitutes reside.”*

Participants highlighted that remaining active during the conflict made them vulnerable to rape and property looting. They couldn't halt work due to food scarcity, leading them to work extensively, which increased the risk of sexual assault. They explained as follows,

"I feared working. There was no freedom. Working during war made us vulnerable to violence.” “They approached whenever they saw smoke.” (L. FGD: p2)

### Severity of rape

The violence's intensity varied based on the number of men involved and the nature of assault. The sexual violence inflicted upon women was distressing. Apart from rape, the actions of the perpetrators were devastating. Raped women and their husbands were arrested and subjected to beatings. Participants detailed the severity of rape as follows:

A participant in in-depth interview mentioned that, “There was a case of a woman being raped for seven men. They targeted not only typical women but also sexually assaulted mentally ill women.”

A participant in in-depth interview mentioned that *“There was a case of a woman being raped by seven men. They targeted not only typical women but also sexually assaulted mentally ill women.”*

One of FGD member explained that*, “We heard of a young girl raped by an HIV-positive person. She tried to escape by claiming she had HIV, but they found someone in the group who was HIV positive and he raped her. Now, she's not just a victim of rape but has also contracted HIV.”*

A 39 years old rape survivor woman explored that “They raped my 14 years old daughter after they tied me and my brother and then they raped me after they tied my daughter and my brother. (Participant 4)

"I was paralyzed by shock and he threatened me with a gun.” (Cried Participant 6)

### Consequences of rape among survivors

Rape profoundly affects women, with significant consequences like psychosocial trauma, stigma, fear of humiliation, divorce, displacement, and economic hardships. Participants detailed these impacts through sub-themes like psychosocial effects, stigma and humiliation, divorce, displacement, and economic consequences. Their explanations are outlined below.

### Psycho social impacts

Women faced significant psychological repercussions post-rape. All victims reported psychological issues such as depression, suicidal attempts, regret, and feeling unwell. Those who disclosed their experiences were affected not only by the assault but also by community stigma, exacerbating their psychological distress.

One rape survivor expressed*, “Rape has caused me mental illness.” Another victim shared, “We suffered psychological distress. Our emotional pain is severe.” A 14-year-old described, “Afterward, I attempted suicide multiple times, but my mother saved me.”*

“I just feel so bad when someone asked about this action. It hurt me a lot” (part 6).

“I tried to lose my life when that problem arose” part (10) “I was saddened” (part 8)

Multiple assaults by different armed groups intensify the mental trauma on women. Moreover, witnessing the beating and torture of their husbands exacerbates their psychological distress, leaving wives in a perpetual state of fear.

"The men had to venture out, facing the risk of being killed. As a result, women suffered severe injuries, endured psychological trauma, and faced physical abuse.” (Participant 1, M. FGD)

### Stigma and fear of humiliation

Women refrained from speaking out due to fear of community norms and stigmatization (*n* = 2).

“*I kept quiet because I didn't want to face shame and stigma…” (p8).*

Eight women disclosed their experience to friends, family, religious figures, and support organizations but later regretted doing so.

A 39 years old woman explored that *“Revealing my experience led to humiliation and added to my psychological distress. People's reactions and the lack of support hurt me deeply”.* (p4: sorrowed).

### Divorce

Women who were previously married ended up divorced after the assault. Single women feared not being able to marry if they revealed the incident. A 25 years old participant expressed that “I didn't marry; I don't know what happens to the future” “A woman, who had hidden her assault for a long time, reached out for support from organizations after getting divorced.” (p6).

A married and 27 years old woman said “But after the attack, my husband was the one who comforted me. He is the one who says I am in side of you”.

Women who were previously married were ended up divorced after the assault. Single women feared not being able to marry if they revealed the incident. A 25 years old participant expressed that *“concerns about the future due to this fear. I didn't marry; I don't know what happens to the future” (p6).*

A married and 27 years old woman said “But after the attack, my husband was the one who comforted me. He is the one who says I am in side of you”.

"A woman, who had hidden her assault for a long time, reached out for support from organizations after getting divorced.” (p6)

### Displacement

Raped women often faced displacement due to community stigma and lack of support. The hostile environment forced them to leave their villages, as they encountered insults and disrespect.

“She received no support, feeling uncomfortable at home; now she’s displaced to another area.” (FGD p5)

### Economic consequences

The conflict severely impacted living conditions, with women experiencing property looting alongside the trauma of rape. Women who are in low income level were forced to work at time of war which predisposed for sexual violence.

“Working during war exposed for violence” (p3: l).

“They can't stop working because there is no food to survive so to meet their need the work as much as possible. I am bearing 2 children without father. To bear them, I sold coffee &tea and can't close during the war. Working during war exposed for violence”. (FGD Women representative).

A 31 years old victim said that “They forced me to bring 5000 birr for buying cigarettes and drink………. When they threaten me by gun I gave them 2500 birr”. (Participant 3: regret) Additionally, one of FGD participant said that “property looting beyond the rapes.”

"A 31-year-old victim was coerced to hand over 2500 birr after being threatened with a gun to fetch 5000 birr for cigarettes and drinks.

Similarly other FGD member explored that “severe attacks during the junta’s arrival, leading to scarcity of resources like water and wood, alongside the distressing fact that their sisters were also raped.”

### Supports for raped women

Medical support: “Victims didn’t receive any medical treatment after the sexual assault.”

Medical support: “Victims didn’t receive any medical treatment after the sexual assault.”

“*I didn*’*t get medical treatment yet”* (p2).

#### Psychological rehabilitation

After the assault, they didn't get any psychological support and were not rehabilitated from the impact.

One of the FGD participant explored that *“Everyone is psychologically harmed. There was no psychological support provided for their psychological harm.”* (FGD p7).

Similarly other FGD member said that *“No psychological support was provided to the victims; they received material aid, but psychological support was absent.” (FGD P2).*

"Despite being raped by seven people, the woman didn't receive psychological support.” (FGD p 3)

## Discussion

The study aimed to identify the extent of sexual violence and its determinants among women, children, and adolescent girls during the Northern Ethiopia conflict. This study found that the magnitude of sexual violence was9.76%, the lower prevalence might stem from underreporting; victims hesitated to report due to fears of divorce, social taboo, and mistreatment. A multi-country study supported our findings, low prevalence of sexual violence was attributed to women's reluctance to report, fearing stigma and its impact on their families, partners, and communities (39). The qualitative finding findings of this study indicated women's hesitation to disclose due to fear of divorce, public gossip, and concerns about being judged for receiving assistance.

This finding contrasts with a study from Mozambique (40), potentially due to varying sample sizes, socio-demographics, and differences in the studied population. Women involved in prostitution showed a higher likelihood of experiencing sexual violence compared to others. This aligns with similar findings from cross-sectional studies conducted in Debre-markos (4) and Ethiopia (41).

This study finding showed that women involved in prostitution showed a higher likelihood of experiencing sexual violence compared to others. This aligns with similar findings from cross-sectional studies conducted in Debre Markos (4) and Ethiopia (41). The finding that women involved in prostitution were more likely to experience sexual violence is further supported by the qualitative component of this study. The qualitative result revealed that the locations where prostitutes lived were identified as higher-risk areas for incidents of sexual violence to occur. The heightened vulnerability of certain exposed groups, such as prostitutes, might push them into risky situations for survival, despite lacking compensation.

Women residing in areas affected by village attacks were three times more likely to experience sexual violence. This vulnerability could stem from heightened exposure to offenders in these locations. Sexual violence was roughly three times more prevalent among women earning 2,001–4,000 ETB monthly (AOR: 2.548) compared to those earning 4,001 or more. This could be linked to the prolonged vulnerability of women with lower incomes, who were actively working to overcome financial constraints. The qualitative findings of the study further support and survivors reported that they were actively working during the conflict period as a coping mechanism, as they did not have previously saved money and were living on a day-to-day limited income. In order to manage this challenge, the women continued working, but this simultaneously made them more prone to experiencing sexual violence and having their property looted. The need to keep working as a coping strategy placed these women in higher-risk situations where they were more vulnerable to such attacks. This could be linked to the prolonged vulnerability of women with lower incomes, who were actively working to overcome financial constraints. Women with higher incomes might have relocated away from conflict areas, while those with lower incomes might have stayed due to financial constraints, increasing their vulnerability to sexual violence. This is echoed in qualitative findings where low-income women were forced to work during the conflict, heightening their risk of violence.

Most incidents occurred at home when the victims were alone. This aligns with studies from the USA and Colombia, where assaults often happened in apartments or residences when individuals were by themselves (7, 42).

Participants experienced significant psychological distress due to sexual violence. Those who disclosed their experience faced emotional repercussions such as depression, suicidal thoughts, and regret, potentially attributed to the severe distress and negative mental health outcomes associated with such assaults, like PTSD and anxiety (43). A study conducted in Iraq found that individuals who experienced sexual violence exhibited high levels of depression and post-traumatic stress disorder (PTSD) symptoms (44).

Many chose not to disclose their experiences, fearing psychological harm and the stigma associated with disclosure in prior research (42). In this study one of the consequences of sexual violence was community stigma. This finding is further supported by a study conducted in Sierra Leone, which showed that victims and survivors of sexual violence often suffer from feelings of shame, fear, ostracism, and distrust within their communities. These experiences can lead them to withdraw from social interactions (15). Similarly, a qualitative study conducted in the Democratic Republic of Congo also found that experiences of rape by armed groups increased the level of stigma faced by survivors (45). Likewise, a study from northeastern Ethiopia indicated that survivors of sexual violence were confronted with a range of stigma and discrimination within their communities (46).

Research at the U.S. Military Academy mirrors this, identifying fear of losing friends and shame as primary reasons for not reporting. Concerns about gossip, self-handling, and fear of blame also played significant roles (47). Similar to a University of Maine study in the USA, women in this study feared losing their partners post-assault, leading to divorce (42). Rape survivors often moved away due to the assault, community stigma, and a sense of humiliation.

### Strength and limitation of the study

The study has strengths of exploring experiences of survivors of sexual violence. Even though the study has strengths, there were some limitations such as the sensitive nature of the issue being investigated, additionally; the research was constrained by limited resources. Time constraints also played a crucial role, as the researchers had to work within a tight schedule. Furthermore, a lack of internet access in certain areas posed a barrier to reaching a wider audience.

## Conclusion

Women, children and adolescent girls were sexually assaulted in North Wollo Zone during the northern conflict. In conclusion this study's findings showed that the magnitude of sexual violence were considerable, an issue that should never be experienced by any woman. This study provides findings from both quantitative and qualitative study. Being prostitute, low income and presence of attacks in the village were significant predictors of sexual violence.

Qualitative results give insights about the condition of event, predisposing factors and its implication on their lives. Survivors were severely traumatized in terms of the number of actors participated in the assault and some were comorbid with HIV as intentionally raped by HIV positive armed persons. Psychosocial, external blame and stigma, fear of humiliation, divorce, displacement and economic hardships were the main impacts of the assault.

## Data Availability

The original contributions presented in the study are included in the article/Supplementary Material, further inquiries can be directed to the corresponding author.
